# Evaluation of Mobile Phone and Cordless Phone Use and Glioma Risk Using the Bradford Hill Viewpoints from 1965 on Association or Causation

**DOI:** 10.1155/2017/9218486

**Published:** 2017-03-16

**Authors:** Michael Carlberg, Lennart Hardell

**Affiliations:** Department of Oncology, Faculty of Medicine and Health, Örebro University, 701 82 Örebro, Sweden

## Abstract

*Objective.* Bradford Hill's viewpoints from 1965 on association or causation were used on glioma risk and use of mobile or cordless phones.* Methods.* All nine viewpoints were evaluated based on epidemiology and laboratory studies.* Results.* Strength: meta-analysis of case-control studies gave odds ratio (OR) = 1.90, 95% confidence interval (CI) = 1.31–2.76 with highest cumulative exposure. Consistency: the risk increased with latency, meta-analysis gave in the 10+ years' latency group OR = 1.62, 95% CI = 1.20–2.19. Specificity: increased risk for glioma was in the temporal lobe. Using meningioma cases as comparison group still increased the risk. Temporality: highest risk was in the 20+ years' latency group, OR = 2.01, 95% CI =1.41–2.88, for wireless phones. Biological gradient: cumulative use of wireless phones increased the risk. Plausibility: animal studies showed an increased incidence of glioma and malignant schwannoma in rats exposed to radiofrequency (RF) radiation. There is increased production of reactive oxygen species (ROS) from RF radiation. Coherence: there is a change in the natural history of glioma and increasing incidence. Experiment: antioxidants reduced ROS production from RF radiation. Analogy: there is an increased risk in subjects exposed to extremely low-frequency electromagnetic fields.* Conclusion.* RF radiation should be regarded as a human carcinogen causing glioma.

## 1. Introduction

In Sir Austin Bradford Hill's classic epidemiology paper from 1965, “*The Environment and Disease: Association or Causation?,*” he warned not to overrate the value of statistical significance since it often leads people to “*grasp the shadow and loose the substance*” of what is in the data [1]. In the interpretation of epidemiological studies on cancer there may be no explanation about how the strength of a link between a cause and an effect can vary from a “*scientific suspicion of risk*” to a “*strong association*” through “*reasonably certainty*” and to “*causality*” which requires the strongest evidence. This continuum in strengths of evidence, which was illustrated in Bradford Hill's paper, written at the height of the tobacco and lung cancer controversy, is not always explained. This means that the media and the public may assume that “*not causal*” means “*no link,*” with mobile phone use and brain tumour risk as one example.

In the Interphone study on mobile phone use and brain tumours an increased risk for glioma was found among the heaviest mobile phone users [2]. In an editorial accompanying the Interphone results published in the International Journal of Epidemiology [3], the main conclusion of the results was described as “*both elegant and oracular… (which) tolerates diametrically opposite readings.”* They also pointed out several methodological reasons why the Interphone results were likely to have underestimated the risks, such as the short latency period since first exposures became widespread; less than 10% of the Interphone cases had more than 10 years of exposure. “*None of the today's established carcinogens, including tobacco, could have been firmly identified as increasing risk in the first 10 years or so since first exposure.*” The concluding sentences from the Interphone study were “*oracular*”*:* “*Overall, no increase in risk of either glioma or meningioma was observed in association with use of mobile phones. There were suggestions of an increased risk of glioma, and much less so meningioma, at the highest exposure levels, for ipsilateral exposures and, for glioma, for tumours in the temporal lobe. However, biases and errors limit the strength of the conclusions we can draw from these analyses and prevent a causal interpretation*.” This allowed the media to report opposite conclusions.

Due to the widespread use of wireless phones (mobile and cordless phones) an evaluation of the scientific evidence on the brain tumour risk was necessary. Thus, in May 2011 the International Agency for Research on Cancer (IARC) at WHO evaluated at that time published studies. The scientific panel reached the conclusion that radiofrequency (RF) radiation from mobile phones, and from other devices, including cordless phones, that emit similar nonionizing electromagnetic field (EMF) radiation in the frequency range 30 kHz–300 GHz, is a Group 2B, that is, a “possible,” human carcinogen [4, 5]. The IARC decision on mobile phones was based mainly on case-control human studies by the Hardell group from Sweden [6–13] and the IARC Interphone study [2, 14, 15]. These studies provided supportive evidence of increased risk for brain tumours, that is, glioma and acoustic neuroma.

No doubt the IARC decision started a worldwide spinning machine to question the evaluation, perhaps similar to the one launched by the tobacco industry when IARC was studying and evaluating passive smoking as a carcinogen in the 1990s [16]. Sowing confusion and manufacturing doubt is a well-known strategy used by the tobacco and other industries [17–19]; see also Walker [20].

A fact sheet from WHO issued in June 2011 shortly after the IARC decision in May 2011 stated that “*to date, no adverse health effects have been established as being caused by mobile phone use”* [21]. This statement contradicted the IARC evaluation and was not based on evidence at that time on a carcinogenic effect from RF radiation and was certainly remarkable since IARC is part of WHO. Furthermore WHO wrote that “*currently, two international bodies have developed exposure guidelines for workers and for the general public, except patients undergoing medical diagnosis or treatment. These guidelines are based on a detailed assessment of the available scientific evidence.” *These organizations were the International Commission on Non-Ionizing Radiation Protection (ICNIRP) and the Institute of Electrical and Electronics Engineers (IEEE).

ICNIRP is a private organization (NGO) based in Germany that selects its own members. Their source of funding is not declared. IEEE is the world's most powerful federation of engineers. The members are or have been employed in companies or organizations that are producers or users of technologies that depend on radiation frequencies, such as power companies, the telecom industry, and military organizations. IEEE has prioritized international lobbying efforts for decades especially aimed at the WHO.

The IARC conclusion was soon also questioned by, for example, some members of ICNIRP [22]. The article by Swerdlow et al. appeared online 1 July 2011, one month after the IARC decision, and concluded that “*the trend in the accumulating evidence is increasingly against the hypotheses that mobile phone use can cause brain tumours in adults.”*

Soon after that other persons affiliated with ICNIRP, Repacholi and associates, made a review on wireless phone use and cancer risks. The paper appeared online October 21, 2011 [23], with similar conclusions as the Swerdlow et al. paper [22].

The exposure guideline by ICNIRP was established in 1998 [24] and was based only on thermal (heating) effects from RF-EMF neglecting nonthermal biological effects. It was updated in 2009 [25] and stated that “*it is the opinion of ICNIRP that the scientific literature published since the 1998 guidelines has provided no evidence of any adverse effects below the basic restrictions and does not necessitate an immediate revision of its guidance on limiting exposure to high frequency electromagnetic fields. …Therefore, ICNIRP reconfirms the 1998 basic restrictions in the frequency range 100 kHz–300 GHz until further notice.”* The guideline still provided by ICNIRP for RF radiation is 2 to 10 W/m^2^ depending on frequency. It should be noted that the ICNIRP guideline is used in most European countries as well as in many other countries. Unfortunately it is based on old data with no acknowledgment of cancer effects or nonthermal biological effects from RF-EMF exposure.

There are a vast number of scientific articles that show nonthermal adverse health effects from RF radiation. These, as well as thermal effects, have been evaluated in several reports. In contrast to ICNIRP the BioInitiative Reports from 2007 [26], updated in 2012 [27], based the evaluation of health hazards also on nonthermal health effects from RF radiation. The BioInitiative 2012 Report, with updated references, defined the scientific benchmark for possible health risks as 30 to 60 *μ*W/m^2^. Considering also chronic exposure and sensitivity among children the precautionary target level was proposed to one-tenth of this, 3–6 *μ*W/m^2^ [27].

The guideline in the BioInitiative Report obviously contradicts the one proposed by ICNIRP. The ICNIRP exposure level has been vigorously propagated by that organization in order to harmonize guidelines worldwide. With few exceptions it has been a successful story and most countries have adopted the ICNIRP guideline. This gives a “green card” to roll out the technology with increasing RF radiation exposure to the population, for example, using wireless Internet access in schools [28], since the high exposure level in the guideline by ICNIRP is rarely compromised. Thus, the exposure target level in the BioInitiative Report is not acknowledged by, for example, the Swedish Radiation Safety Authority (SSM). Many persons at the SSM expert panel are also members of ICNIRP which might be a conflict of interests since they would rarely compromise the ICNIRP view; critical opinions are not heard. As a matter of fact the Ethical Board at the Karolinska Institute in Stockholm, Sweden, concluded already in 2008 that being a member of ICNIRP may be a conflict of interests that should be stated in scientific publications (Karolinska Institute Diary Number 3753-2008-609), which is however not done to our knowledge.

An association between use of wireless phones and glioma has not been acknowledged by several scientific bodies in spite of the IARC classification in May 2011. This is exemplified below. In fact, as can be seen in Table 1 the same persons may appear in different expert groups. This would hardly make any substantial difference in the opinion between these groups. They may in fact cite themselves by claiming that various organizations have come to similar conclusion. It is striking how ICNIRP has infiltrated the WHO Monograph core group making it less likely that the conclusions in that Monograph will differ from ICNIRP's conclusions.

*ICNIRP (2011)*. See conclusions at page 1537 [22].

The limited duration of data yet available, which is mainly for up to 10 years of exposure and to a lesser extent for a few years beyond this, also leave uncertainty because of the potential for long lag period effects, especially for meningioma which is generally slower growing than glioma. The possibility of a small or a longer term effect thus cannot be ruled out. Nevertheless, while one cannot be certain, the trend in the accumulating evidence is increasingly against the hypothesis that mobile phone use causes brain tumours.

*AGNIR; Health Protection Agency (2012)*. See conclusions at page 312 [29].

In conclusion, despite methodological shortcomings, the available data do not suggest a causal association between mobile phone use and fast growing tumours such as malignant glioma in adults.

*Exposure from Mobile Phones, Base Stations, and Wireless Networks: A Statement by the Nordic Radiation Safety Authorities (2013)*. See page 1 [30].

The overall data published in the scientific literature to date do not show adverse health effects from exposure of radiofrequency electromagnetic fields below the guidelines or limits adopted in the Nordic countries…Since 2011, a number of epidemiological studies on mobile phone use and risk of brain tumours and other tumours of the head have been published. The overall data on brain tumour and mobile phone use do not show an effect on tumour risk.

*Health Canada (2015) [31].*

*Myth: The International Agency for Research on Cancer (IARC) classified radiofrequency energy as potentially carcinogenic. This means that I will get cancer due to my exposure to RF energy.*

*Fact: The IARC did not find a direct link between RF energy exposure and cancer.*

*In 2011, the International Agency for Research on Cancer (IARC), which is part of the World Health Organization, classified radiofrequency electromagnetic fields as possibly carcinogenic to humans (Group 2B), based on an increased risk for glioma, a malignant type of brain cancer, associated with wireless phone use. However, the vast majority of research to date does not support a link between RF energy exposure and cancers in humans. *

*IET: The Institution of Engineering and Technology, UK (2014; Updated 2016)*. See page 2 [32].

BEPAG has concluded in this report that the balance of scientific evidence to date does not indicate that harmful effects occur in humans due to low-level exposure to EMFs. Our examination of the peer-reviewed literature published in the last two years has not justified a change in the overall conclusions published in our previous report in May 2014.

*SCENIHR: Scientific Committee on Emerging and Newly Identified Health Risks (2015)*. See page 84 [33].

Overall, the epidemiological studies on RF EMF exposure do not indicate an increased risk of brain tumours, and do not indicate an increased risk for other cancers of the head and neck region, or other malignant diseases including childhood cancer.

And looking further down at the same page, we find the following.

A working group at the International Agency for Research on Cancer (IARC) within the Monograph programme on the evaluation of carcinogenic risks to humans classified the epidemiological evidence for glioma and acoustic neuroma as limited and therefore evaluated RF fields as a possible human carcinogen (IARC, 2013). Based on studies published since that assessment (update of the Danish cohort study, the UK cohort study, further case-control studies, the case-control study on mobile phones and brain tumours in children and adolescents, the consistency checks of brain tumour incidence rates using data from the Nordic countries and the US), the evidence for glioma has become weaker.

*SSM: Swedish Radiation Safety Authority (2015)*. See page 6 [34].

However, in previous reports the Scientific Council of SSM has concluded that studies of brain tumours and other tumours of the head (vestibular schwannoma, salivary gland), together with national cancer incidence statistics from different countries, are not convincing in linking mobile phone use to the occurrence of glioma or other tumours of the head region among adults. Recent studies described in this report do not change this conclusion although these have covered longer exposure periods. Scientific uncertainty remains for regular mobile phone use for time periods longer than 15 years.

*Health Council of the Netherlands Mobile Phones and Cancer, Part 3. Update and Overall Conclusions from Epidemiological and Animal Studies (2016)*. See page 54 [35].

The available data do not allow drawing conclusions on whether there is an association between an increased carcinogenic risk and any form of accumulation of exposure, for instance expressed in the total call time, or the total amount of energy deposited by the electromagnetic fields generated by the phone in the head or in any other body part…. However, it is possible that some individuals would like to reduce their exposure, despite the conclusion of the Committee that there is no consistent evidence for an increased risk for tumours in the brain and other regions in the head associated with mobile phone use.

*SSM: Swedish Radiation Safety Authority (2016)*. See page 91 [36].

Regarding mobile phone use and brain tumour risk, little new data was published and several papers deal with reanalyses of already published data. As a consequence, little has changed in the rating of the evidence.

It should be noted that SSM has never acknowledged an increased risk for brain tumours associated with use of wireless phones.

We published in 2013 an article on using the Bradford Hill viewpoints for brain tumour risk and use of wireless phones [37]. We concluded that based on these aspects “*glioma and acoustic neuroma should be considered to be caused by RF-EMF emissions from wireless phones and regarded as carcinogenic to humans.”* Since then the scientific literature in this area has expanded considerably. Furthermore, as exemplified above, after the IARC evaluation in May 2011, several committees have evaluated the evidence on health risks associated with use of mobile phones. It should also be noted that these reports are not published in the peer-reviewed scientific literature and few physicians if at all are members of these groups. There seems also to be conflict of interests among these members. It is thus pertinent to make a new scientific evaluation using the Bradford Hill viewpoints including the most recent publications.

## 2. Methods

Sir Austin Bradford Hill used nine viewpoints in his President's Address on circumstances that may “*pass from observed association to a verdict of causation”* [1]. Our research group has for long time studied RF radiation and health risks. This has included continuous surveillance of data bases on new studies, especially PubMed, but also personal communications with updated references from other researchers in this area. This article is partly based on our previous publication [37] and a presentation at the Royal Society of Medicine, London, October 13, 2016.

Statistical methods to calculate odds ratios (OR) and 95% confidence intervals (CIs), to visualize risks using restricted cubic splines, and to calculate trends in incidence using joinpoint regression analysis, are presented in our different cited articles. Random-effects model was used for all meta-analyses using StataSE 12.1 (Stata/SE 12.1 for Windows; Stata Corp., College Station, TX, USA).

## 3. Results

### 3.1. Strength

The first viewpoint discussed by Hill is strength of an association.  Table 2 gives results for highest cumulative use in hours for mobile phones. Note that in our study cordless phones were included in the wireless category [38]. The highest group of cumulative use in Coureau et al. [39] was ≥896 h (10th percentile) as compared to Interphone [2] ≥1,640 h (10th percentile). The results in Hardell and Carlberg [38] were recalculated using the same category for highest cumulative exposure as in Interphone [2]. The meta-analysis yielded OR = 1.90, 95% CI = 1.31–2.76. The results are consistent with a statistically significant increased risk for glioma. The study by Turner et al. [40] was a reanalysis of only parts of the Interphone data and was not included in this meta-analysis.

Strength of association is also supported by a 5-country study as part of Interphone on glioma risk in relation to estimated RF brain tumour dose from mobile phones [15]. In case-case analyses comparing tumours in the highest exposed area with tumours located elsewhere the OR for glioma in the highest exposed area was highest in the group with longest use, 10+ years, yielding OR = 2.80, 95% CI = 1.13–6.94.

In a case-control study on brain tumours among patients aged 7–19 years at the time of diagnosis an elevated risk was found based on operator recorded use of mobile phone; OR = 2.15, 95% CI = 1.07–4.29 in the longest latency group >2.8 years [41]. The result was based on only 24 exposed cases and 25 exposed controls. Type of brain tumour was not reported among these cases.

### 3.2. Consistency

Similar results should be found by different research groups and in different populations. The Interphone study group included 13 different countries, whereas Coureau et al. [39] covered four areas in France and Hardell and Carlberg [38] covered Sweden. It should be noticed that there was no overlapping of subjects between our studies and the Swedish part of Interphone. In these three different studies there is a consistent finding of increased glioma risk increasing with latency, Table 3. The highest OR was found with the longest latency, 10+ years. Meta-analysis gave OR = 1.62, 95% CI = 1.20–2.19 in the longest latency group. The result was based on 732 exposed cases and 1,279 exposed controls.

Inclusion criteria for cases and controls differed between Interphone [2] and our study [38]. Thus we included subjects 20–80 years in contrast to Interphone including the age group 30–59 years. Furthermore Interphone disregarded use of cordless phones in contrast to our studies assessing use of wireless phones: mobile phones and cordless phones. We analyzed our material in the age group 30–59 years and included use of cordless phones in the “unexposed” group in our study for the time period 1997–2003 [10]. This yielded similar results for glioma in both studies, for example, in Interphone ipsilateral cumulative mobile phone use ≥1,640 h OR = 1.96, 95% CI = 1.22–3.16 and in our study OR = 2.18, 95% CI = 1.09–4.35; contralateral use OR = 1.25, 95% CI = 0.64–2.42 and OR = 1.48, 95% CI = 0.57–3.87, respectively. Similar results were also found for glioma in the temporal lobe; see Table 2 in Hardell et al. [42].

### 3.3. Specificity

Specificity deals with specific exposure and particular sites and types of the disease. Here we analyze only RF radiation. According to one study the temporal lobe is mostly exposed during use of the handheld mobile phone; see Table 4 [43]. Thus, highest glioma risk would be expected for tumours in the temporal lobe.

Table 5 shows highest risk in the temporal lobe in studies from three research groups. Results are given for the highest group of cumulative use for glioma in the temporal lobe with similar findings.

As also discussed above under Strength, Cardis et al. [15] gave results for glioma in the highest exposed area of the brain based on estimated RF radiation dose. OR increased with time since start of mobile phone use yielding highest risk in the 10+ latency group.

The Interphone study included 13 countries during the study period 2000–2004. The major results were published after a delay of 6 years in 2010 [2]. In a new publication the intracranial distribution of glioma in relation to RF radiation from mobile phones was analyzed [44]. Tumour localization for 792 regular mobile phone users was analyzed in relation to distance from preferred ear for mobile phone use. Five categories for the distance were used with ≥115.01 mm as the reference category (*α* = 1.0). The *α* values represent the change in risk of observing a tumour within the given interval in comparison with the baseline intensity. An association with distance from preferred side of mobile phone use to center of tumour was found; the closer the distance, the higher the risk. The highest risk was found in the group with the closest distance (0–55 mm) yielding *α* = 2.37, 95% CI = 1.56–4.56. Tumour size, duration of phone use, cumulative phone use, and cumulative number of calls were analyzed. Although no statistically significant differences were found overall, higher risks with decreasing distance were found in the upper levels of these dichotomized covariates; see Table 6 showing results for the shortest distance group (0–55 mm).

Our case-control studies included all types of brain tumours reported to the Swedish Cancer Register regardless of tumour type [38]. Assessment of exposure was used without knowledge of tumour type. We found no consistent evidence of increased risk for meningioma associated with use of wireless phones. In one analysis, meningioma cases (*n* = 1,624) were used as the reference entity to glioma cases (*n* = 1,379).  Table 7 shows a statistically significant increased risk for glioma associated with ipsilateral use of all phone types. Ipsilateral mobile phone use gave OR = 1.40, 95% CI = 1.06–1.84, and ipsilateral cordless phone OR = 1.42, 95% CI = 1.06–1.90. Thus the association between RF radiation and brain tumour risk was specific for glioma.

### 3.4. Temporality

The temporal association between exposure and disease is important. Both initiation and promotion/progression of the disease are of relevance.  Table 8 shows highest risk in the group with longest latency (time from first exposure to disease). Our study is the only one with results for latency >20 years for wireless phone use yielding OR = 2.01, 95% CI = 1.41–2.88. Also Interphone [2] and Coureau et al. [39] showed increasing risk with latency.

**Figure 1:**
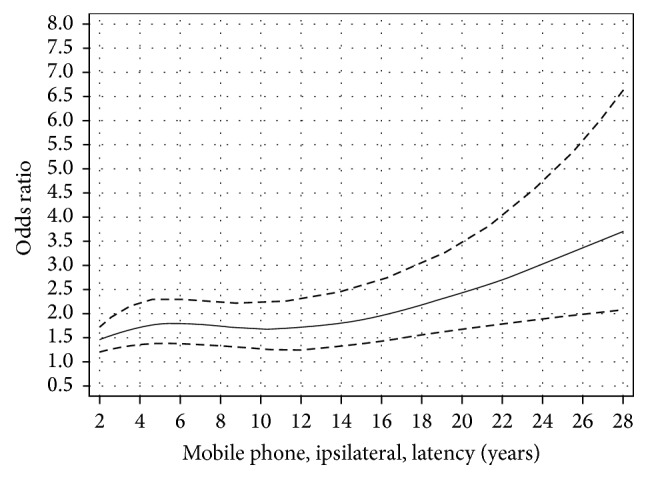
Restricted cubic spline plot of the relationship between latency of ipsilateral mobile phone use and glioma. The solid line indicates the OR estimate and the broken lines represent the 95% CI. Adjustment was made for age at diagnosis, gender, socioeconomic index (SEI), and year for diagnosis. Population based controls were used [38].

**Figure 2:**
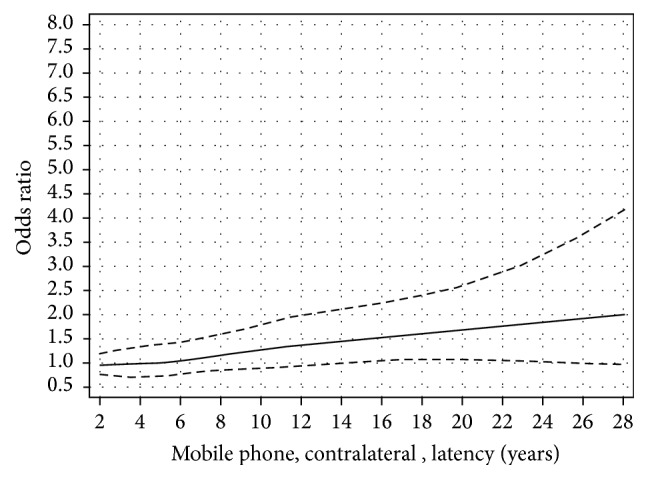
Restricted cubic spline plot of the relationship between latency of contralateral mobile phone use and glioma. The solid line indicates the OR estimate and the broken lines represent the 95% CI. Adjustment was made for age at diagnosis, gender, socioeconomic index (SEI), and year for diagnosis. Population based controls were used [38].

**Figure 3:**
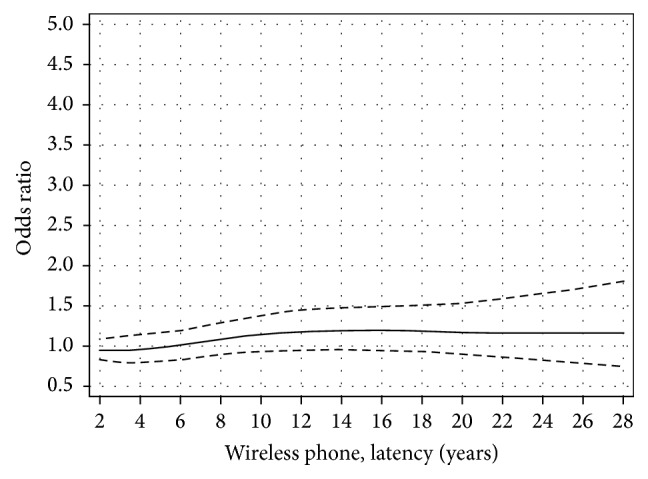
Restricted cubic spline plot of the relationship between latency of wireless phones and meningioma. The solid line indicates the OR estimate and the broken lines represent the 95% CI. Adjustment was made for age at diagnosis, gender, socioeconomic index (SEI), and year for diagnosis.

We used restricted cubic splines to visualize the relationship between latency and cumulative ipsilateral use of mobile phone. The results for latency and ipsilateral mobile phone use show that there was an increased OR with short latency and after some decline an increasing risk with longer latency (nonlinearity, *p* = 0.01); see Figure 1 [38]. This finding is different from the result for contralateral mobile phone use; see Figure 2 (nonlinearity, *p* = 0.76). The results were similar for cordless phone use, data not in figures (ipsilateral, nonlinearity, *p* = 0.04; contralateral, nonlinearity, *p* = 0.26). It should be noted that contralateral use was defined as >50% use on the opposite side of the head compared to the tumour localization. Thus, in spite of being coded as contralateral exposure some ipsilateral tumour exposure (less than 50%) may have occurred. These results are in contrast to meningioma risk with OR close to unity regardless of latency, Figure 3; see also Specificity above.

### 3.5. Biological Gradient

Sir Bradford Hill mentioned that if the association shows a biological gradient, dose-response, it should be more carefully considered. Interphone [2] included 2,708 cases and 2,972 controls in the analysis. In the last decile of cumulative exposure ≥1,640 h a statistically significant increased risk for glioma was found, OR = 1.40, 95% CI = 1.03–1.89; see Table 9. In the other categories of cumulative use a decreased risk was found. Bias and confounding were discussed as potential reasons for that. Analyzing only subjects with regular use of a mobile phone yielded OR = 1.82, 95% CI = 1.15–2.89 in the group with highest cumulative use. There was an age difference between cases and controls in the Interphone material and furthermore cases and the matched controls were interviewed at different time periods, controls usually later than cases. This is problematic for mobile phones with rapid penetration of the use in the population. In an alternative analyses cases and controls nearest in age and time for interview were included [40]. The association between mobile phone use and glioma was strengthened thereby. Thus among regular users in the 10th decile (≥1,640 h) cumulative use gave OR = 2.82, 95% CI = 1.09–7.32.

Also Coureau et al. [39] found highest risk in the highest group of cumulative use, ≥896 h, with OR = 2.89, 95% CI = 1.41–5.93 with a statistically significant trend (*p* = 0.02); see Table 10. The results were based on 253 participating cases and 504 participating controls.

We divided cumulative use in hours of wireless phones into quartiles. The results were based on 1,380 responding glioma cases and 3,530 responding controls [38]. For both mobile and cordless phones the highest risk was found in the fourth quartile with a statistically significant trend; see Table 11.

### 3.6. Plausibility

One aspect on association or causality is if the disease is biologically plausible. The IARC evaluation in May 2011 [4, 5] concluded that there is “*limited evidence in experimental animals for the carcinogenicity of radiofrequency radiation”*; see page 419 [5].

Effects on tumour susceptibility in mice exposed to a UMTS (universal mobile telecommunications system) test signal from fetal time for up to 24 months were studied by Tillmann et al. [45]. Animals were exposed to UMTS fields with intensities of 0, 4.8, and 48 W/m^2^. The low-dose group, 4.8 W/m^2^, was subjected to additional prenatal ethylnitrosourea (ENU) treatment. The ENU-treated group and UMTS-exposed at 4.8 W/m^2^ showed an increased lung tumour rate and an increased incidence of lung carcinomas as compared to the controls treated with ENU only. The authors concluded that the study showed a cocarcinogenic effect of lifelong UMTS exposure in female mice subjected to pretreatment with ENU. This study was included in the IARC evaluation.

The results by Tillmann et al. [45] gained further interest based on the results in a follow-up study published in 2015 [46]. The exposure levels were 0 (sham), 0.04, 0.4, and 2 W/kg SAR. Numbers of tumours of the lungs and livers and malignant lymphoma in exposed animals were statistically significant higher than in sham-exposed controls. A tumour-promoting effect from RF radiation was found at low to moderate levels (0.04 and 0.4 W/kg SAR), well below exposure limits for users of mobile phones [46].

A report was released from The National Toxicology Program (NTP) under the National Institutes of Health (NIH) in USA on the largest ever animal study on cell phone RF radiation and cancer [47]. An increased incidence of glioma in the brain and malignant schwannoma in the heart was found in rats. Acoustic neuroma or vestibular schwannoma is a similar type of tumour as the one found in the heart, although benign. These results have gained considerable interest since epidemiological human studies have in addition to glioma also found an increased risk for acoustic neuroma, also called vestibular schwannoma [48].

In a study published in 2013 exposure to 1,800 MHz RF radiation induced oxidative DNA base damage in a mouse spermatocyte-derived cell line [49]. There was a concomitant increase in reactive oxygen species (ROS). This effect was mitigated by cotreatment with the antioxidant *α*-tocopherol. The authors concluded that RF radiation with insufficient energy for the direct induction of DNA strand breaks can give genotoxicity through oxidative DNA base damage.

Yakymenko et al. [50] showed in a review of 100 studies investigating oxidative effects of low-intensity RF radiation in living cells that exposure down to 2,500 *μ*W/m^2^ [51] and with SAR values down to 600 *μ*W/kg [52, 53] could increase oxidative stress in the cells. It should be noted that the guideline for mobile phone SAR is 2 W/kg. The 2 W/kg guideline is in any 10 g of tissue while in the US (FCC guideline) the exposure limit value is 1.6 W/kg in 1 g of tissue. Certainly these results on oxidative stress are of concern since ROS are of crucial importance in carcinogenesis.

### 3.7. Coherence

Hill points out that an association would be strengthened if an exposure changes the biology and natural history of the disease. One interesting gene is the* p53* protein. It is a transcription factor that plays a vital role in regulating cell growth, DNA repair, and apoptosis, and* p53 *mutations are involved in disease progression. In a study it was found that use of mobile phones for ≥3 hours a day was associated with increased risk for the mutant type of* p53 *gene expression in the peripheral zone of glioblastoma multiforme, the most malignant glioma type. Furthermore, this mutation increase was statistically significant correlated with shorter overall survival time [54].

We analyzed survival of 1,678 glioma patients in our 1997–2003 and 2007–2009 case-control studies [55]. Use of wireless phones in the >20 years' latency group (time since first use) gave a reduced survival yielding hazard ratio (HR) = 1.68, 95% CI = 1.23–2.29 for glioma; see Table 12. For glioblastoma multiforme (high-grade glioma; *n* = 926) mobile phone use yielded HR = 1.99, 95% CI = 1.37–2.91 and cordless phone use HR = 3.37, 95% CI = 1.04–11 in the same latency category. The hazard ratio for glioblastoma multiforme increased statistically significant per year of latency for wireless phones, HR = 1.020, 95% CI = 1.007–1.033, and of borderline statistical significance per 100 h cumulative use, HR = 1.002, 95% CI = 0.999–1.005. The hazard ratio was highest in the age group <20 years for first use of a wireless phone; see Table 13.

In contrast for low-grade astrocytoma (grades I-II; *n* = 228) decreased HR (increased survival) was found for mobile phone use HR = 0.50, 95% CI = 0.29–0.88 and cordless phone use HR = 0.60, 95% CI = 0.34–1.07, and for wireless phones in total statistically significant decreased HR = 0.57, 95% CI = 0.34–0.94. The reason for the survival benefit for cases with astrocytoma grades I-II associated with use of both mobile and cordless phones is unclear. However, surgery is crucial for survival in patients with low-grade astrocytoma that may transform to high-grade glioma in the long term. An earlier treatment gives a better prognosis. Tumour promotion from RF radiation might give earlier symptoms leading to surgery. For 144 (63%) of the 228 cases with low-grade astrocytoma it was possible to calculate tumour volume based on CT/MRI scans [55]. This gave for cases exposed to wireless phones (*n* = 121) median volume = 25.1 cm^3^ (mean = 37.7, range = 0.15–179.6) compared with unexposed cases (*n* = 23) median volume = 18.3 cm^3^ (mean = 33.1, range = 0.79–125.7). Although the difference was not statistically significant (*p*, Wilcoxon rank-sum test = 0.82), these results indicate tumour promotion from RF radiation since the median tumour volume was 37.2% larger in exposed cases. This might cause tumour awareness and earlier surgery.

Also for glioblastoma multiforme the median tumour volume was larger in exposed cases (*n* = 346) than in unexposed cases (*n* = 112), 25.6 versus 22.0 cm^3^, that is, 16.4% larger volume,* p*, Wilcoxon rank-sum test = 0.68 [55]. This tumour type is extremely malignant with median survival in the range of 6 months in spite of surgery and radio- and chemotherapy [56]. Thus early detection does not significantly change the prognosis.

**Figure 4:**
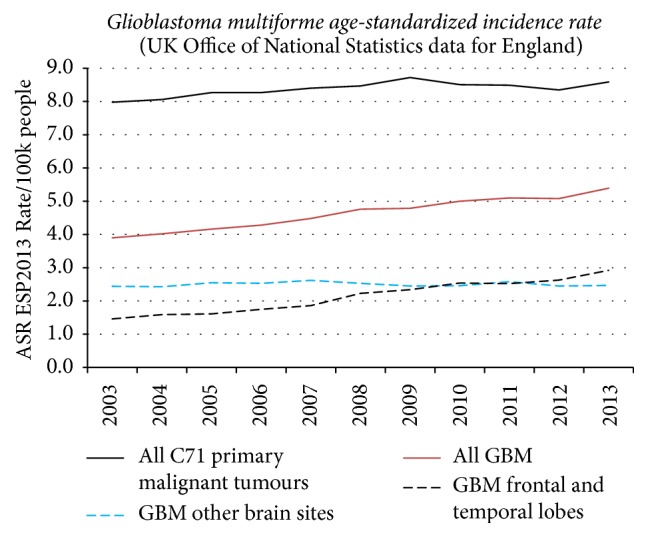
Graphical data on age-standardized incidence rate of glioblastoma multiforme in England 2003–2013. Data provided by Alasdair Philips. A detailed analysis is under publication.

It has been suggested that overall incidence data on brain tumours for countries may be used to qualify or disqualify the association between mobile phones and brain tumours observed in case-control studies. During recent years such opinions have been published by different study groups. However, it must be stressed that descriptive epidemiology with no individual exposure data is of less value than results in analytical epidemiology such as case-control studies. Studies should primarily be aimed at investigating incidence in the most exposed part of the brain, the temporal lobe. Of special interest is the incidence of the most malignant glioma type, glioblastoma multiforme. We have discussed this in previous publications, for example, [37, 57]. In England increasing incidence of glioblastoma multiforme, especially in the frontal and temporal lobes, during 2003–2013 has been found; see Figure 4. Of interest is that a real increase in the incidence of glioblastoma multiforme in frontal and temporal lobes and cerebellum was reported in USA [58].

**Figure 5:**
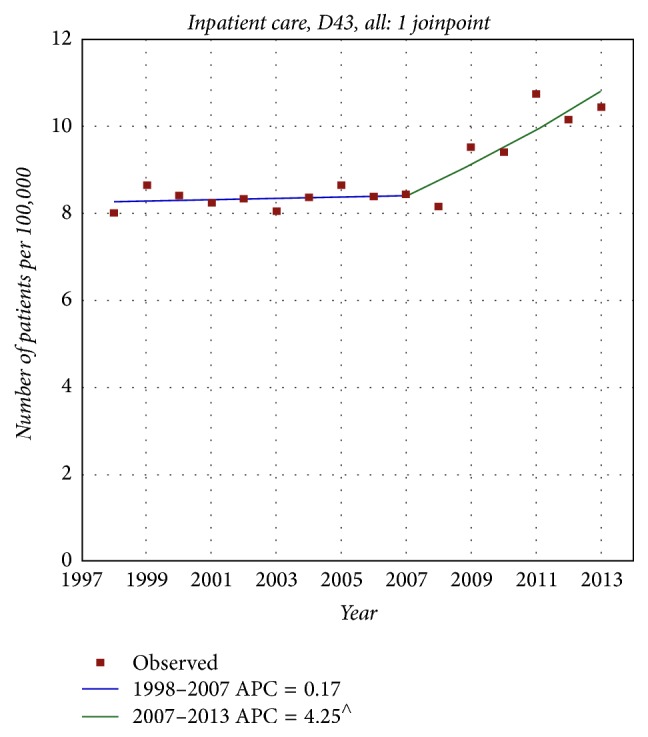
Joinpoint regression analysis of number of patients per 100,000 inhabitants according to the Swedish National Inpatient Register for both genders combined, all ages during 1998–2013 diagnosed with D43 = tumour of unknown type in the brain or CNS [59]. ^∧^Statistically significant trend.

**Figure 6:**
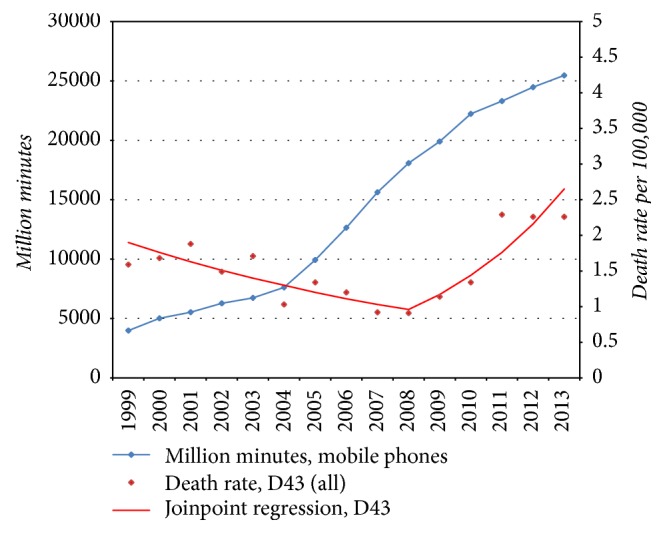
Number of outgoing mobile phone minutes in millions during 1999–2013 and joinpoint regression analysis of age-standardized death rates per 100,000 inhabitants according to the Swedish Causes of Death Register for all ages during 1999–2013 diagnosed with D43 = tumour of unknown type in the brain or CNS [59].

No increasing incidence of brain tumours has been recorded in the Swedish Cancer Register. We have discussed the many shortcomings in the reporting of new cases elsewhere [59]. Using the Swedish Inpatient Register (IPR) we found an increasing rate of patients with D43 = tumour of unknown type in the brain or CNS with joinpoint in 2007; see Figure 5. A joinpoint was found in 2008 for increasing death rate of D43 in the Swedish Causes of Death Register, Figure 6. No histopathology is available for these cases but they may represent glioblastoma multiforme based on results in IPR with joinpoint in 2007 and the short survival for these patients.

In an ecological study from England annual incidence of brain tumours in the temporal and parietal lobes was modelled based on population-level covariates. The study period was 1985–2014. Malignant brain tumours in the temporal lobe increased faster than would be expected. Using a latency period of 10 years this increase was related to the penetration of mobile phone use. This corresponded to an additional increase of 35% (95% credible interval 9%; 59%) or 188 (95% CI 48–324) additional cases annually [60]. The author concluded that the findings were in agreement with mobile phones and other wireless equipment being causing factors.

### 3.8. Experiment

Sir Bradford Hill discussed in his paper if prevention has an effect on the risk. Relating to wireless phones no such community experiment exists. Antioxidants such as melatonin, vitamin C, and vitamin E (*α*-tocopherol) may alleviate the generation of ROS [49, 61]. There are however no studies if persons taking antioxidants and using wireless phones have a reduced risk for glioma.

Mobile phones were introduced in Sweden in the early 1980s. First, it was very common to use the phone in a car with external antenna without any use outside the car. In our first study period 1997–2000 a number of cases and controls had only used the mobile phone in a car with external antenna. In addition one control reported always use of a hands-free device [8]. They were regarded as unexposed to RF radiation. Brain tumour risk in this group was calculated to crude OR = 0.82, 95% CI = 0.59–1.15.

### 3.9. Analogy

The last viewpoint by Bradford Hill is analogy. Is there some evidence with another similar exposure? One analogy would be glioma risk associated with extremely low-frequency electromagnetic fields (ELF-EMF). In 2002 IARC classified ELF-EMF as “possibly carcinogenic to humans,” Group 2B based on an increased risk for childhood leukemia [62]. More recently a pooled analysis showed about twofold increased risk for childhood leukemia at exposure level above 0.3–0.4 *μ*T [63], further supporting a carcinogenic potential from ELF-EMF.

ELF-EMF is generated by alternating electric currency and humans may be exposed both during leisure time and in different occupational settings. In an evaluation of epidemiological findings on exposure to ELF-EMF it was concluded regarding glioma that an increased risk was seen in electric and electronics industries [64].

Based on occupational history it was possible to calculate ELF-EMF job exposure for cases and controls using a job-exposure matrix (JEM) both in Interphone [65] and in our studies [66].

In the international Interphone study glioma was associated with occupational ELF-EMF exposure in recent time windows whereas no increased risk was found for meningioma [65]. The authors concluded that such exposure may play a role in late stage carcinogenesis of glioma.

The results in our studies were based on 1,346 glioma cases and 3,485 population based controls [66]. Cumulative exposure (≥90th percentile versus <25th percentile) increased the risk for glioblastoma multiforme in 5-year time windows (data not in table) up to 14 years; see Table 14 for time windows 1–14 and 15+ years.

With longer latency periods (15+ years) no statistically significant increased risk and trend were found. For low-grade glioma no statistically significant increased risk was seen in the different time windows. In conclusion this study showed an increased risk in late stage (promotion/progression) of glioblastoma multiforme for occupational ELF-EMF exposure.

## 4. Discussion

In this review we considered all nine viewpoints by Bradford Hill on association or causation regarding use of wireless phones and glioma risk. It is an update of our article from 2013 on this issue [37] since more scientific evidence has emerged since then. As discussed above after the IARC evaluation in 2011 concluding RF radiation to be “possibly carcinogenic” to humans several organizations have stated that the association has been weaker or even no consistent evidence for an increased risk for brain tumours. This has in part been based on a much criticized Danish cohort study on persons with mobile phone subscriptions and assumed mobile phone use with funding from the telecom industry [67]. The study was not based on sound epidemiological principles and had several methodological limitations mainly due to poor exposure assessment that render it to be uninformative at best [68]. Some of the many shortcomings include the following.

1. Corporate subscribers of mobile phones (200,507 people), which are likely to have been heavy users, were classified as “unexposed.”
2. Mobile phone subscription holders not using the phone were classified as “exposed.”
3. Users of cordless phones not using a mobile phone were classified as “unexposed.”
4. Nonsubscribers using the mobile phone were classified as “unexposed.”
5. Persons with a mobile phone subscription later than 1995 were classified as “unexposed.”
6. No individual exposure data were assessed (e.g., on cumulative exposure or side of head mostly used).
7. No operator-verified data on years of subscription were assessed.

These limitations are likely to have led to an underestimate of any risk in this study. One would expect considerable misclassification of mobile phone use both among subscribers and the reference population since no new subscribers were included in the exposed cohort after 1995. We stated that “*after reviewing the four publications on the Danish cohort study, one might rightly wonder whether this cohort was initially set up to show no increased risk.”* A similar conclusion was made by IARC in the 2011 evaluation, thus stating that using the “*reliance on subscription to a mobile-phone provider, as a surrogate for mobile phone use, could have resulted in considerable misclassification in exposure assessment*” [4]. The Danish cohort study should no longer be cited as scientific evidence on no increased risk for glioma among mobile phone users.

A study in UK published in 2013 has been included in the* no risk* paradigm [69]. Use of mobile phones was assessed in about 65% of a cohort of women established for other purposes during 1996–2001. Only baseline data collected at one time between 1999 and 2005 were used with the questions: “*About how often do you use a mobile phone?” *(never, less than once a day, every day) and “*For how long have you used one?”* (total years of use). In 2009, the participants were asked how much they did talk on a mobile phone and how many years they had used the phone. However, these later data were not used in the analysis. Of those reporting no use of a mobile phone at baseline, 49% reported such use in 2009. The incidence of brain tumours was assessed in 2005 and the average follow-up was only 7 years. No increased incidence of glioma was found (*n* = 571 cases). For acoustic neuroma (*n* = 96 cases), there was an increase in risk with long term use versus never use (10+ years: relative risk (RR) = 2.46, 95% CI = 1.07–5.64, *p* = 0.03), the risk increasing with duration of use (trend among users, *p* = 0.03). No data were available on handedness for mobile phone use or tumour localization in the brain. Use of cordless phones was ignored. This study had poor assessment of exposure and has the same shortcomings as the Danish cohort study. Benson et al. gave in a letter to the Editor updated follow-up data to 2011 [70]. They found no longer a statistically significant increased risk for acoustic neuroma. However, these results were based on the same baseline data as previously and similarly lack scientific precision in the assessment of exposure. Due to the many shortcomings this study should not be cited as evidence of no increased risk for glioma among mobile phone users.

Not all are careful in the evaluation of scientific evidence on RF radiation and glioma risk. Repacholi et al. in their article published on line 2011 included the Danish cohort study in the review on glioma risk [23]. They stated that they included also cordless phone use although no results were presented from the German part of Interphone claimed to have assessed cordless phone use. We have found in our studies a consistent increased glioma risk associated with use of cordless phones [38]. However, Repacholi et al. stated that “*most of the studies from the Hardell group report an association whereas other studies do not. The reason for this is unclear.” *One reason is that the other studies like Interphone did not report use of cordless phones thus diminishing the risk towards unity [71]. In fact, the results in the Hardell group studies are similar to Interphone and Coureau et al.; see Tables 2, 3, 5, and 8. Repacholi et al. considered the Hill viewpoints thereby excluding some of the viewpoints and modifying others. They concluded that “*in summary, none of the Hill criteria support a causal relationship between wireless phone use and brain cancers or other tumors in the areas of the head that most absorb the RF energy from wireless phones. Accordingly, the conclusions and recommendations of WHO [2011] provide adequate protective measures, and the ICNIRP guidelines limiting exposure to RF fields [ICNIRP, 1998, 2009b] continue to provide a sound, science-based standard for public health policy regarding the use of wireless phones by adults.” *Obviously this conclusion is not based on an understanding and thorough evaluation of Hill's viewpoints. At best it might be an example of misunderstanding scientific evidence without basic knowledge in pathology and oncology. The practice to misuse Hill's viewpoints (misinterpreted as criteria for causation) has been discussed by Kundi [72].

In contrast to the Repacholi et al. publication [23] we have used the original Hill viewpoints without modification or exclusions. That would give a more decent and true evaluation based on these viewpoints. Regarding* strength* Hill wrote that “*we must not be too ready to dismiss a cause-and-effect hypothesis merely on the ground that the observed association appears to be slight.*” Our analysis showed doubled risk for glioma in the group with highest cumulative exposure; see Table 2. Thus similar results were found in different populations by different study groups.

Regarding* consistency*, Bradford Hill wrote that the observed association has been “*repeatedly observed by different persons, in different places, circumstances and times.” *As can be seen in Table 3 consistency was found not only for cumulative use but also for latency.

*Specificity* is a “*strong argument in favour of causation*” according to Hill. Ipsilateral exposure to RF radiation in the temporal lobe is the area with highest exposure to RF radiation. There is a consistent finding of increased risk for use of the wireless phone on the same side as the tumour occurred. This risk is confirmed in analysis of glioma risk in the temporal lobe, and also using distance to the mobile phone and estimated total cumulative specific energy in J/kg [15]. Furthermore the risk is specific for glioma using meningioma cases as the comparison group in the same study [38].

The* temporal* relationship of the association is important. Thus, exposure should precede the disease outcome. In carcinogenesis also latency (time from exposure to glioma diagnosis) is of relevance. Clearly OR increased with latency in the case-control studies with highest risk in the 20+ group [38]. The maximum latency was shorter in Interphone [2] and Coureau et al. [39] but still yielded highest risk.

A* biological gradient*, dose-response, should be found. In the case-control studies a statistically significant trend with increasing call time in hours was reported by Coureau et al. [39] and in our study [38]. In Interphone a statistically significant increased risk was only found in the 10th decile of cumulative use ≥1,640 hours. Also restricting the analysis to subjects with regular mobile phone use gave highest risk in the same group, OR = 1.82, 95% CI = 1.15–2.89. No trend analysis was reported; see Appendix 2 [2]. In the alternative post hoc matching of cases and controls in Interphone (closest in age and time for interview) the 10th decile of cumulative use gave OR = 2.82, 95% CI = 1.09–7.32 [40].

For* plausibility* Hill stated that “*it will be helpful if the causation we suspect is biologically plausible. But this is a feature I am convinced we cannot demand. What is biologically plausible depends upon the biological knowledge of the day.*” By now there are studies showing a cocarcinogenic and tumour-promoting effect from RF radiation. One postulated mechanism would be generation of ROS that can give base-pair damage of DNA. These effects have been shown in several experimental studies with RF radiation levels well below current guideline for exposure during use of mobile phones.

For* coherence* the natural history and biology of the disease are evaluated. One interesting aspect is the increased risk for the mutant type of the* p53* gene expression in glioblastoma multiforme associated with use of mobile phones [54]. The mutation is involved in disease progression and shorter survival was found in patients with the mutant gene. This finding is of large interest in relation to our result showing shorter survival in patients using mobile or cordless phones [55]. The age group <20 years for first use of the wireless phone had the highest hazard ratio, that is, the strongest reduction in survival. The tumour volume was larger in glioma cases using wireless phones compared with nonusers. It should also be noted that *α* was higher in larger glioma tumours with shortest distance from preferred ear to tumour center which might be an effect of tumour promotion [44]. Several studies have shown an increasing incidence of glioma, especially glioblastoma multiforme in the temporal lobe. These facts show a change in the natural history of the disease.

It is difficult to perform an* experiment *for a rare disease like glioma. Thus, the risk would be studied among persons that have stopped use of wireless phones and analyze a possible risk reduction over time as seen for lung cancer risk in ex-smokers. Such a cohort study is in practice almost impossible to perform, especially for a rare disease like brain tumour. Some indirect evidence might be found by the finding in our study that use of mobile phone in a car with external antenna and no other use of a wireless phone (no exposure to RF radiation) gave no increased brain tumour risk [8]. This finding, as well as the alleviation of ROS production from RF radiation by antioxidants, might be proxies for experiment.

The last viewpoint by Hill is* analogy*. Is there glioma risk with similar exposure? ELF-EMF has been classified as* possibly human carcinogen*, Group 2B by IARC in 2002 [62]. Based on occupational ELF-EMF exposure an increased risk for glioma has now been found in two case-control studies [65, 66].

## 5. Conclusion

The nine Bradford Hill viewpoints on association or causation regarding RF radiation and glioma risk seem to be fulfilled in this review. Based on that we conclude that glioma is caused by RF radiation. Revision of current guidelines for exposure to RF radiation is needed.

## Figures and Tables

**Table 1:** Members of WHO Monograph core group and their involvement in different other groups.

Name	WHO	ICNIRP	UK/AGNIR	SSM	SCENIHR
Simon Mann	X	X	X		
Maria Feychting	X	X	X	X^*∗*^	
Gunnhild Oftedal	X	X			
Eric van Rongen	X	X		X	
Maria Rosaria Scarfi	X	X^*∗*^		X	X
Denis Zmirou	X				

^*∗*^Former.

WHO: World Health Organization.

ICNIRP: International Commission on Non-Ionizing Radiation Protection.

AGNIR: Advisory Group on Non-Ionising Radiation.

SSM: Strålsäkerhetsmyndigheten (Swedish Radiation Safety Authority).

SCENIHR: Scientific Committee on Emerging and Newly Identified Health Risks.

**Table 2:** *Strength*. Numbers of cases (Ca), controls (Co), and odds ratio (OR) with 95% confidence interval (CI) for glioma in case-control studies in the highest category of cumulative use in hours for mobile phone use.

	Ca/Co	OR	95% CI
*Interphone 2010*			
Cumulative use ≥ 1,640 h	210/154	1.40	1.03–1.89
*Coureau et al. 2014*			
Cumulative use ≥ 896 h	24/22	2.89	1.41–5.93
*Hardell and Carlberg 2015*			
Cumulative use ≥ 1,640 h	211/301	2.13	1.61–2.82
*Turner et al. 2016 (Interphone)*			
Cumulative use ≥ 1,640 h	59/46	2.82	1.09–7.32
*Meta-analysis* ^*∗*^			
Cumulative use ≥ 1,640 h^*∗∗*^	445/477	1.90	1.31–2.76

^*∗*^Based on Interphone [2], Coureau et al. [39], and Hardell and Carlberg [38].

^*∗∗*^≥896 h used for Coureau et al.

Random-effects model used for all meta-analyses, based on test for heterogeneity in the overall group (“all mobile”).

**Table 3:** *Consistency*. Numbers of cases (Ca), controls (Co), and odds ratio (OR) with 95% confidence interval (CI) for glioma and latency in three different case-control studies, Interphone 2010 (mobile phone) [2], Coureau et al. 2014 (mobile phone) [39], and Hardell and Carlberg 2015 (wireless phones) [38].

	Interphone Appendix 2		Coureau et al. 2014		Hardell and Carlberg 2015		Meta-analysis	
	Ca/Co	OR (95% CI)	Ca/Co	OR (95% CI)	Ca/Co	OR (95% CI)	Ca/Co	OR (95% CI)
Unexposed^*∗*^	93/159	(1.0) —	107/219	(1.0) —	306/1,058	(1.0) —	506/1,436	(1.0) —
*Latency*								
2–4 years^*∗∗*^	460/451	1.68 (1.16–2.41)	49/122	0.88 (0.56–1.39)	215/575	1.17 (0.95–1.44)	724/1,148	1.22 (0.90–1.66)
5–9 years	468/491	1.54 (1.06–2.22)	66/111	1.34 (0.87–2.06)	339/799	1.27 (1.05–1.54)	873/1,401	1.33 (1.13–1.55)
10+ years	190/150	2.18 (1.43–3.31)	22/31	1.61 (0.85–3.09)	520/1,098	1.39 (1.15–1.67)	732/1,279	1.62 (1.20–2.19)

^*∗*^Unexposed Interphone Appendix 2: latency 1–1.9 years; unexposed in Coureau et al.: not regular user; unexposed Hardell and Carlberg: no use + latency ≤ 1 year.

^*∗∗*^1–4 years in Coureau et al.

**Table 4:** *Specificity*. Distribution of average specific absorption rate (SAR): %.

	900 MHz	1,800 MHz
Right		
(i) Brainstem	1	0.2
(ii) Cerebellum	12	13
(iii) Frontal	19	14
(iv) Occipital	5	5
(v) Parietal	9	7
(vi) Temporal	50	60
Total	96.8%	99.5%
Left	3.2%	0.5%

Based on Cardis et al. [43].

**Table 5:** *Specificity*. Numbers of cases (Ca), controls (Co), and odds ratio (OR) with 95% confidence interval (CI) for glioma and all mobile phone use and in the temporal lobe for mobile phone use ≥ 1,640 h, in three different case-control studies [2, 38, 39].

	Interphone 2010		Coureau et al. 2014		Hardell, Carlberg 2015	
All	1,666/1,894	0.81 (0.70–0.94)	142/270	1.24 (0.86–1.77)	945/2,148	1.31 (1.09–1.58)
Temporal lobe, ≥1,640 h	78/47	1.87 (1.09–3.22)	7/5^*∗*^	3.94 (0.81–19.08)	59/301	2.05 (1.36–3.10)

^*∗*^≥896 h.

**Table 6:** *Specificity*. Estimated elevation in brain tumour risk (*α*) and 95% confidence interval (CI) in shortest distance group 0–55 mm compared to reference category ≥ 115.01 mm from preferred ear to tumour center. Based on Grell et al. [44].

Covariate	*α*	95% CI
Tumour size		
≤18 cm^3^	1.96	1.51–3.66
>18 cm^3^	4.09	1.90–12.0
Duration of phone use		
<6 years	2.02	1.31–4.28
≥6 years	3.27	1.92–11.3
Cumulative phone use		
<200 hours	1.57	1.29–3.36
≥200 hours	4.06	2.03–11.6
Cumulative number of calls		
<4,000	1.55	1.25–3.42
≥4,000	3.56	2.05–9.88

**Table 7:** *Specificity*. Odds ratio (OR) and 95% confidence interval (CI) for glioma (*n* = 1,379) and meningioma cases (*n* = 1,624) as the reference entity. Numbers of exposed cases (Ca) and controls (Co) are given. Adjustment was made for age at diagnosis, gender, socioeconomic index (SEI), and year for diagnosis [38].

	Ipsilateral			Contralateral		
	Ca/Co	OR	95% CI	Ca/Co	OR	95% CI
Analogue	190/106	1.69	1.15–2.47	98/75	1.25	0.78–2.00
Digital (2G)	549/432	1.46	1.09–1.96	298/329	1.00	0.71–1.40
Digital (UMTS, 3G)	35/26	3.27	1.21–8.83	21/17	1.79	0.61–5.20
Mobile phone, total	591/458	1.40	1.06–1.84	316/342	1.02	0.74–1.41
Cordless phone	461/378	1.42	1.06–1.90	258/289	1.11	0.79–1.56

**Table 8:** *Temporality*. Odds ratio (OR) and 95% confidence interval (CI) for latency and glioma risk in three different case-control studies, Interphone 2010 (mobile phone) [2], Coureau et al. 2014 (mobile phone) [39], and Hardell and Carlberg 2015 (wireless phones) [38].

	All	>10 y latency	>20 y latency
*Interphone (2010)*			
Mobile phone	0.81 (0.70–0.94)	2.18^*∗*^(1.43–3.31)	—
*Coureau et al. (2014)*			
Mobile phone	1.24 (0.86–1.77)	1.61 (0.85–3.09)	—
(i) Last decile of cumulative duration (≥896 h)			
(a) 1 y censorship	2.89 (1.41–5.93)		
(b) 5 y censorship	5.30 (2.12–13.23)		
*Hardell and Carlberg (2015)*			
Wireless phone	1.31 (1.11–1.56)	1.55 (1.23–1.96)	2.01 (1.41–2.88)
Mobile phone	1.31 (1.09–1.58)	1.55 (1.21–1.99)	2.18 (1.50–3.15)

^*∗*^Regular use, 1–1.9 y latency = OR 1.0.

**Table 9:** *Biological Gradient*. Numbers of cases (Ca), controls (Co), and odds ratio (OR) with 95% confidence interval (CI) for cumulative use of mobile phone in hours and glioma risk in Interphone [2].

Cumulative call time (hours)	Ca/Co	OR (95% CI)
Never regular user	1,042/1,078	(1.0)
<5	141/197	0.70 (0.52–0.94)
5–12.9	145/198	0.71 (0.53–0.94)
13–30.9	189/179	1.05 (0.79–1.38)
31–60.9	144/196	0.74 (0.55–0.98)
61–114.9	171/193	0.81 (0.61–1.08)
115–199.9	160/194	0.73 (0.54–0.98)
200–359.9	158/194	0.76 (0.57–1.01)
360–734.9	189/205	0.82 (0.62–1.08)
735–1,639.9	159/184	0.71 (0.53–0.96)
≥1,640	210/154	1.40 (1.03–1.89)

≥1,640^*∗*^	160/113	1.82 (1.15–2.89)

^*∗*^Regular use < 5 h = OR 1.0 (ref).

**Table 10:** *Biological Gradient*. Numbers of cases (Ca), controls (Co), and odds ratio (OR) with 95% confidence interval (CI) for cumulative duration of calls (hours) in Coureau et al. [39].

Cumulative duration of calls (hours)	Ca/Co	OR (95% CI)
No use	107/211	(1.0)
<43	24/63	0.83 (0.48–1.44)
43–112	20/55	0.77 (0.42–1.41)
113–338	28/58	1.07 (0.60–1.90)
339–895	28/37	1.78 (0.98–3.24)
≥896	24/22	2.89 (1.41–5.93)

*p* value global test		0.02

**Table 11:** *Biological Gradient*. Numbers of cases (Ca), controls (Co), and odds ratio (OR) with 95% confidence interval (CI) for cumulative duration of calls (hours) of wireless phones in quartiles in Hardell and Carlberg [38].

Quartile	Mobile phone	Cordless phone	Wireless phone
	OR, CI (Ca/Co)	OR, CI (Ca/Co)	OR, CI (Ca/Co)
First quartile, all	1.27 1.05–1.54 (340/920)	1.09 0.86–1.39 (174/478)	1.15 0.94–1.42 (223/641)
Second quartile, all	1.29 1.02–1.65 (198/492)	1.23 0.97–1.57 (203/534)	1.29 1.04–1.61 (235/596)
Third quartile, all	1.36 1.04–1.78 (179/416)	1.64 1.28–2.11 (210/451)	1.37 1.08–1.72 (249/617)
Fourth quartile, all	2.22 1.68–2.92 (228/320)	2.35 1.78–3.10 (165/261)	2.04 1.61–2.57 (367/618)

*p*, trend	<0.0001	<0.0001	<0.0001

First quartile 1–122 h; second quartile 123–511 h; third quartile 512–1,486 h; fourth quartile > 1,486 h.

**Table 12:** *Coherence*. Hazard ratio (HR) and 95% confidence interval (CI) for survival of patients with glioma and use of wireless phones, study period 1997–2009 [55].

Latency > 20 years	Glioma		Glioblastoma multiforme	
	HR	95% CI	HR	95% CI
Wireless phone	1.68	1.23–2.29	2.09	1.45–3.01
Mobile phone	1.79	1.30–2.47	1.99	1.37–2.91
Cordless phone	1.35	0.49–3.72	3.37	1.04–10.92

**Table 13:** *Coherence*. Hazard ratio (HR) and 95% confidence interval (CI) for survival of patients with glioblastoma multiforme and use of wireless phones in different age groups, study period 1997–2009 [55].

	Mobile phone			Cordless phone			Wireless phone		
	*n*, exp	HR	95% CI	*n*, exp	HR	95% CI	*n*, exp	HR	95% CI
*Glioblastoma multiforme (n = 926)*									
*Age, first use*									
<20 years old	10	2.24	1.04–4.85	6	1.78	0.68–4.67	11	2.27	1.10–4.71
20–49 years old	296	1.24	0.98–1.58	177	1.31	1.001–1.72	328	1.23	0.99–1.53
≥50 years old	226	1.11	0.91–1.36	232	1.09	0.88–1.34	279	1.14	0.95–1.37

**Table 14:** *Analogy*. Odds ratio (OR) and 95% confidence interval (CI) for glioblastoma multiforme for occupational exposure to ELF-EMF in time windows; 1–14 years and 15+ years before diagnosis. Unconditional logistic regression, adjusted for age at diagnosis, gender, socioeconomic index (SEI), and year of diagnosis. Exposure the year before diagnosis was excluded (“1-year lag”). Numbers of exposed cases (Ca) and controls (Co) are given [66].

Cumulative exposure (*µ*T-years)	Glioblastoma multiforme (*n* = 687)		
	Ca/Co	OR	95% CI
*1–14 years' time window*			
<0.91	106/770	1.00	—
0.91–<1.42	138/872	1.28	0.96–1.70
1.42–<1.82	187/778	1.79	1.36–2.35
1.82–<2.75	129/537	1.81	1.35–2.43
2.75+	89/329	1.88	1.35–2.62
*p, linear trend*			<0.001
*15+ years' time window*			
<1.44	119/782	1.00	—
1.44–<2.55	154/777	1.05	0.80–1.39
2.55–<4.17	173/787	0.99	0.74–1.32
4.17–<6.59	126/471	0.93	0.66–1.30
6.59+	88/313	0.91	0.62–1.32
*p, linear trend*			0.44
